# Special Issue “Advanced Pulse Laser Machining Technology”

**DOI:** 10.3390/ma16020819

**Published:** 2023-01-14

**Authors:** Jörg Krüger, Jörn Bonse

**Affiliations:** Bundesanstalt für Materialforschung und -prüfung (BAM), Unter den Eichen 87, D-12205 Berlin, Germany

“Advanced Pulse Laser Machining Technology” is a rapidly growing field that can be tailored to special industrial and scientific applications. This is significantly driven by the availability of high-repetition-rate laser sources and novel beam delivery concepts. In recent publications, Saraceno et al. [1], Schille and Löschner [2], and Weber and Graf [3] presented graphs of the development of ultrashort-pulse laser technology over the past few decades (see the synthesis of data from [2,3] provided in Figure 1). Obviously, the average power of ultrafast lasers follows a type of Moore’s law, leading to the doubling of the average power of these lasers every two years [4]. Additionally, the average power attained by the lasers in research laboratories precedes the average power of standard industrial lasers by about ten years [3], visualized here as the horizontal separation between the two lines in Figure 1. The impressive progress in laser technology currently culminates in the availability of a fiber-laser-based average power of 10.4 kW at a 1.4 µm wavelength, 254 fs pulse duration, and 80 MHz repetition rate [5]. Currently, the industrial standard is ultrashort-pulse lasers emitting average powers of the order of 100 W.

For industrial use, the high output power of ultrafast lasers must be directed to the workpieces to be machined by appropriate beam guidance and deflection systems [6]. For many materials such as metals and semiconductors, moderate laser fluences (i.e., laser pulse energies) are sufficient to process the workpiece with high precision and efficiency [7,8,9]. Therefore, high laser pulse repetition rates up to the above-mentioned MHz level can be utilized to significantly reduce the processing times. This requires extremely fast beam deflection systems, possibly coupled with optics for multibeam processing. In a recent study, an area processing rate up to 3.8 m^2^/min was demonstrated with a single-pass raster on steel sheets with a scanning speed of the laser beam of 950 m/s [10]. The key element of the experimental setup was a polygon scanner [11] in connection with a MHz laser system.

The Special Issue addresses not only the advantages of modern laser processing using short and ultrashort laser pulses, but also limitations caused by unwanted secondary hazards such as X-ray emissions. This phenomenon is not completely new and was described for laser machining applications using ultrashort laser pulses with repetition rates of the order of 1 kHz two decades ago [12,13,14]. However, the use of high pulse repetition rates in the multi-100 kHz range and burst pulses has recently exacerbated the problem [15,16,17,18,19,20,21]. Therefore, it is a pleasant fact that five publications in this Special Issue discuss this problem area in detail and, thus, make an important contribution to the field of combined laser and radiation protection for improving work safety aspects [22,23,24,25,26].

The use of laser pulse bursts enables new process regimes for metals and allows for an increase in the structuring rates and surface quality of machined samples. Results of both experimental and numerical investigations in this context are reviewed by Förster et al. [27]. The machining strategies using pulse bursts with intraburst repetition frequencies in the MHz up to GHz regime must consider an increased risk of secondary X-ray emissions with this mode of operation [21,25,26].

This Special Issue bundles together 1 review paper [27], 1 perspective article [28], and 14 original research articles [22,23,24,25,26,29,30,31,32,33,34,35,36,37], all focusing on the latest achievements in areas of surface and volume laser material processing, including laser-induced forward transfer and laser printing technologies [28], spatial and temporal beam shaping [29,30,31,32], Bessel-beam structuring of high-aspect-ratio void channels inside glass [34], direct laser interference patterning [35], pulse burst machining [27], waveguide writing [36], fs-pulse laser-induced amorphization and recrystallization of single-crystalline silicon [37], and a comparison of different beam shuttering technologies [33]—an aspect that is often neglected, but most practically relevant.

## Figures and Tables

**Figure 1:**
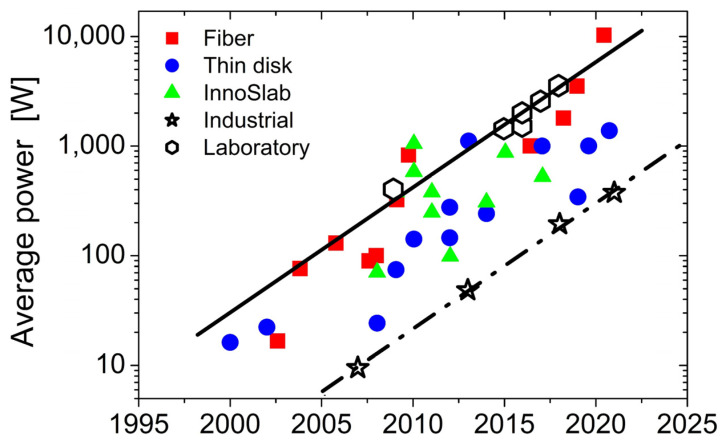
Progress in ultrafast laser technology featuring an exponential increase in the average output power over the past 20 years. The plot is a synthesis of data, indicating the related laser technology (fiber, thin disk, and InnoSlab) as fully colored data points [2], as well as their realization in laboratories or as commercial industrial products as open black data points [3]. The black lines guide the eye.

## Data Availability

Not applicable.
